# Metabolites of Cannabis Induce Cardiac Toxicity and Morphological Alterations in Cardiac Myocytes

**DOI:** 10.3390/ijms23031401

**Published:** 2022-01-26

**Authors:** Ayse Orme Merve, Pola Sobiecka, Vytautas Remeškevičius, Luke Taylor, Lili Saskoy, Scott Lawton, Ben P. Jones, Ahmed Elwakeel, Francesca E. Mackenzie, Elena Polycarpou, Jason Bennett, Brian Rooney

**Affiliations:** 1 School of Life Sciences, Pharmacy and Chemistry, Kingston University, Penrhyn Road, Kingston upon Thames, London KT1 2EE, UK; k1807597@kingston.ac.uk (A.O.M.); k1717476@kingston.ac.uk (P.S.); k1501503@kingston.ac.uk (V.R.); k1328615@kingston.ac.uk (L.T.); k1700497@kingston.ac.uk (L.S.); s.p.lawton@kingston.ac.uk (S.L.); k2060250@kingston.ac.uk (B.P.J.); F.Mackenzie@kingston.ac.uk (F.E.M.); E.Polycarpou@kingston.ac.uk (E.P.); 2 Centre for Sport, Exercise and Life Sciences (CSELS), Coventry University, Pharmacology and Therapeutics, Alison Gingell Building, Whitefriars Street, Coventry CV1 2DS, UK; elwakeela@uni.coventry.ac.uk (A.E.); ad2619@coventry.ac.uk (J.B.)

**Keywords:** cannabis, THC, cardiac myocytes, cytoskeleton, planaria, cardiac toxicity

## Abstract

Cannabis is one of the most commonly used recreational drugs worldwide. Rrecent epidemiology studies have linked increased cardiac complications to cannabis use. However, this literature is predominantly based on case incidents and post-mortem investigations. This study elucidates the molecular mechanism of Δ9-tetrahydrocannabinol (THC), and its primary metabolites 11-Hydroxy-Δ9-THC (THC-OH) and 11-nor-9-carboxy-Δ⁹-tetrahydrocannabinol (THC-COOH). Treatment of cardiac myocytes with THC-OH and THC-COOH increased cell migration and proliferation (*p* < 0.05), with no effect on cell adhesion, with higher doses (250–100 ng/mL) resulting in increased cell death and significant deterioration in cellular architecture. Conversely, no changes in cell morphology or viability were observed in response to THC. Expression of key ECM proteins α-SMA and collagen were up-regulated in response to THC-OH and THC-COOH treatments with concomitant modulation of PI3K and MAPK signalling. Investigations in the planarian animal model *Polycelis nigra* demonstrated that treatments with cannabinoid metabolites resulted in increased protein deposition at transection sites while higher doses resulted in significant lethality and decline in regeneration. These results highlight that the key metabolites of cannabis elicit toxic effects independent of the parent and psychoactive compound, with implications for cardiotoxicity relating to hypertrophy and fibrogenesis.

## 1. Introduction

Cannabis sativa, commonly known as marijuana is one of the most widely used recreational drugs and has elicited significant interest within medical research for the treatment of neuropathic pain, epilepsy, and seizures [1,2,3,4,5,6,7,8]. In recent years, attitudes towards cannabis have evolved, owing to the large-scale decriminalization of medical and recreational cannabis use in countries such as the United States and Canada. The effect of such legislation changes has seen increased cannabis use among younger populations [9].

Δ9-tetrahydrocannabinol (THC) is the main psychoactive constituent of cannabis amongst 500 identified phytocannabinoids [10]. THC interacts with the endogenous endocannabinoid system (ECS), an extensive network of receptors regulating heart rate and blood pressure, along with endocrine and immune responses [9,11,12,13,14,15,16]. The ECS comprises of the cannabinoid-1 and cannabinoid-2 receptors (CB1 and CB2) which are activated by the endogenous endocannabinoids, anandamide and 2-arachidonoylglycerol [17]. THC is a partial agonist to the CB1 receptor, following administration, THC is metabolised through oxidation into the active metabolite 11-Hydroxy-Δ9-THC (THC-OH). 11-OH-THC is further oxidised into a second pharmacologically inactive metabolite, 11-nor-9-carboxy-Δ⁹-tetrahydrocannabinol (THC-COOH) [18,19]. A study by Anis and colleagues identified THC as a mediator of cell cycle arrest, cell apoptosis, inhibition of cell migration and a modulator of F-actin integrity in urothelial cell carcinoma [10]. F-actin is a crucial and abundant protein of the cell cytoskeleton, essential for cellular function, stability and morphogenesis [20]. Response to injury or inflammation activates a cascade of reparative processes which result in changes to the cell cytoskeleton; reciprocation to micro-environmental changes include actin severing and assembly, therein promoting cell migration. Several studies have linked cannabis consumption in otherwise healthy patients with adverse cardiovascular complications such as acute coronary syndrome (ACS), myocardial infarction (MI) and arrhythmia [21,22,23,24]. A predictable effect of cannabis consumption is a 20–100% increase in heart rate and resultant elevation of blood pressure due to vasodilation associated with THC [25]. At higher doses, an increased heart rate is observed partially due to the peripheral inhibition of the parasympathetic system. Similarly, elevated heart rate is often attributed to the pathogenesis of ACS [24].

ACS refers to a wide range of clinical conditions including myocardial injury. During myocardial injury, excess deposition of the extracellular matrix (ECM) occurs in order to preserve the functionality of the left ventricle (LV) as it undergoes molecular, cellular and physiological changes [26]. Fibrosis is defined as an excessive and uncontrolled deposition of ECM proteins, while cardiac fibrosis is characterized by cardiac fibroblast activation and differentiation into myofibroblasts coupled with elevated collagen type I deposition [26]. Collagen I, III, and alpha smooth muscle actin (α-SMA) along with other key matrix proteins commonly deposited the site of the injury, promoting wound repair while simultaneously causing stiffness of the myocardium and increasing risks of future heart failure [27,28]. Alpha Smooth Muscle Actin (αSMA), fibronectin, collagen I and III are the key markers of cardiac fibrosis alongside phospho-AKT and phospho-ERK, protein kinases involved in cell proliferation which inhibit apoptosis and promote cell survival [29,30,31,32].

Recent studies indicate that cannabis abuse can induce cardiovascular and cerebrovascular complications [22,23,24]. In 2019/20, 29.6% of individuals from England and Wales between 16 and 59 used cannabis at least once in their lifetime, an increase of 6%, compared to 2001 to 2002 [33]. A study by Jouanjus, Lapeyre-Mestre and Miallef [34] suggested a link between cannabis and cardiovascular complications with particular risk of ACS and MI. Investigations by Drummer et al. [35] analysed drug overdoses and demonstrated an association between cardiovascular complications and cannabis which, in serious cases, can be the primary cause of death if the condition remains untreated. A review by Thanvi and Treadwell [36] concluded that cannabis abuse, predisposes individuals to stroke and is a contributory factor in ischemic events in 15–40% of cases in young individuals. The bulk of research into cannabis and cardiovascular complications has focused on the pharmacokinetics and pharmacodynamics of cannabinoids, most notably THC. However, the effects exerted by THC-OH and THC-COOH on the microstructural changes of the cardiac architecture have not been extensively researched. The current study investigated the effect of THC and its metabolites in in vitro conditions on cardiac myocytes and in *Polycelis Nigra Planaria* animal model to elucidate the role of cannabinoids on fibrogenesis. Our results indicate that the major metabolites of cannabis, THC-OH and THC-COOH induce morphological changes in the cytoskeleton, hypermotility coupled with increased wound healing and ECM deposition. Higher doses were shown to result in severe cellular toxicity and loss of structural integrity.

## 2. Results

### 2.1. Metabolites of THC Mediate Increased Cell Migration and Wound Closure in Cardiac Cells

**Figure 1:**
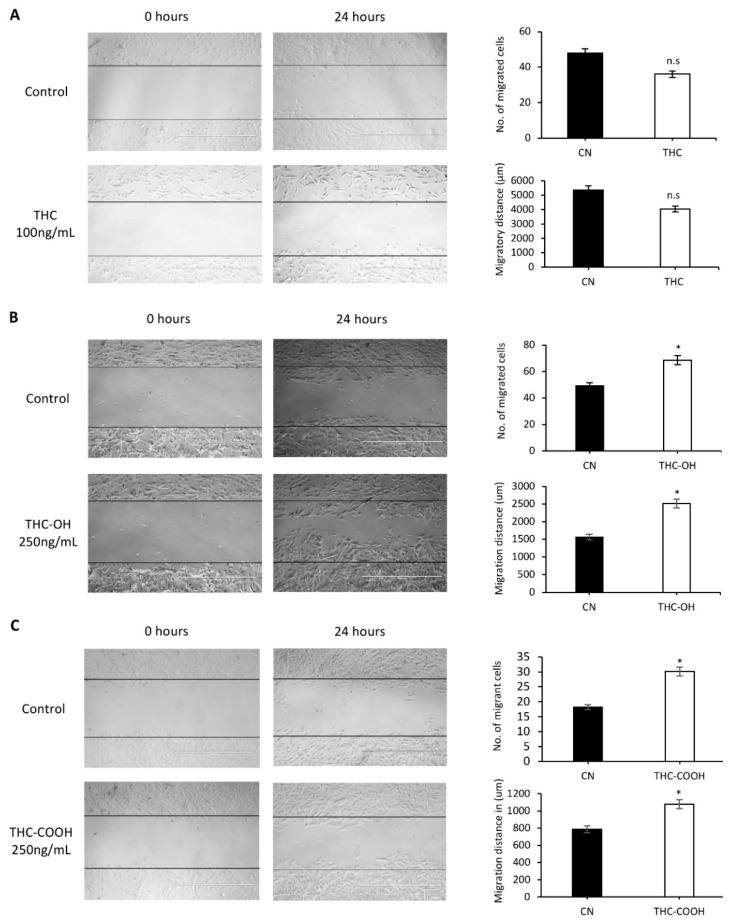
Metabolites of cannabis induce increased wound closure (**A**) H9c2 cardiomyocytes were exposed to 100 ng/mL of THC for 24 h, (**B**) 250 ng/mL of THC-OH for 24 h and (**C**) 250 ng/mL of THC-COOH for 24 h compared with complete growth medium control following scratch wound treatments. The number of migrant cells were quantified by ImageJ analysis software and compared against untreated control cells. The migratory distance of cardiomyocytes in terms of wound healing was determined manually by equivalence of the scale bar and compared with untreated control cells (scale bar was 1000 µm). Statistical comparison for treated vs. control was performed by a two-tailed Students unpaired *t*-test (* *p* < 0.05). n.s. refers to not statistically significant. Results represent the mean of three individual experiments (N = 3).

Treatment with 100 ng/mL THC did not produce a statistically significant increase in cell migration or migration distance when compared to control. Conversely, THC-OH and THC-COOH treatments of 250 ng/mL potentiated cell migration into the wound space within 24-h. Additionally, H9c2 cardiomyocyte migration distance was significantly increased (*p* < 0.05) in THC-OH and THC-COOH treated cells compared to control (Figure 1A–C). These results suggest that the major active psychoactive component of cannabis, THC, does not alter cytoskeletal dynamics but that its short and long-acting metabolites, THC-OH and THC-COOH, promote alterations in cellular morphology and motility.

### 2.2. Cannabinoids Promote Alterations in Cell Proliferation in H9c2 Cells

**Figure 2:**
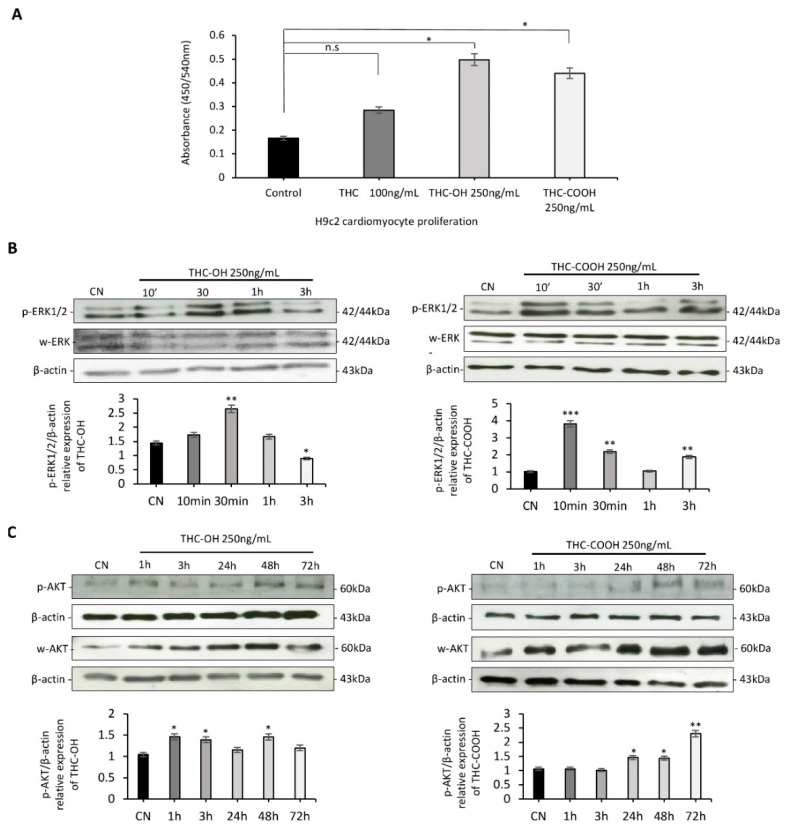
H9c2 changes in cell proliferation in response to cannabinoids. (**A**) H9c2 cardiomyocyte proliferation in response to THC, THC-OH and THC-COOH, represented data of five individual experiments. (**B**) Immunoblot analysis of ERK1/2 phosphorylation following THC-OH and THC-COOH (both 250 ng/mL) exposure. (**C**) Immunoblot analysis of AKT phosphorylation following THC-OH and THC-COOH (both 250 ng/mL) exposure. (**B**,**C**) Representative immunoblots of three independent experiments. Densitometry indicating relative band expression for each blot measured using ImageJ software. Statistical comparison for treated vs. control was performed by a two-tailed Students unpaired *t*-test (n.s. refers to not statistically significant, * *p* < 0.05, ** *p* < 0.01 and *** *p* < 0.001) for individual time points.

A BrdU incorporation assay was used to assess the proliferative potential of cardiomyocytes over a 24-h period. H9c2 cardiomyocytes were treated with 100 ng/mL of THC, 250 ng/mL of THC-OH and 250 ng/mL of THC-COOH (Figure 2A). Statistical comparison performed using a two-tailed Student’s unpaired *t*-test showed no change in the proliferative potential of THC treated cardiomyocytes. Conversely, cardiomyocytes exposed to THC-OH and THC-COOH presented a statistically significant increase (*p* < 0.05) in cell proliferation. Both THC-OH and THC-COOH mediate time dependent increases in expression of ERK1-p44 (Thr-202/Tyr204) and ERK2-p42 (Thr-185/Try-187), within 30 min of 250 ng/mL treatment as shown by western blotting (Figure 2B). These same treatments resulted in an increased phosphorylation of AKT between 48–72 h (Figure 2C). Results from proliferation assays and immunoblot experiments suggest metabolites of THC may act as mediators of increased cell proliferation via a combination of AKT/ERK in H9c2 cardiomyocytes with possible application to fibrogenesis.

### 2.3. Bioinformatic Analysis of Binding Affinities between Key Regulators of Cytoskeletal Dynamics and THC Metabolites

**Figure 3:**
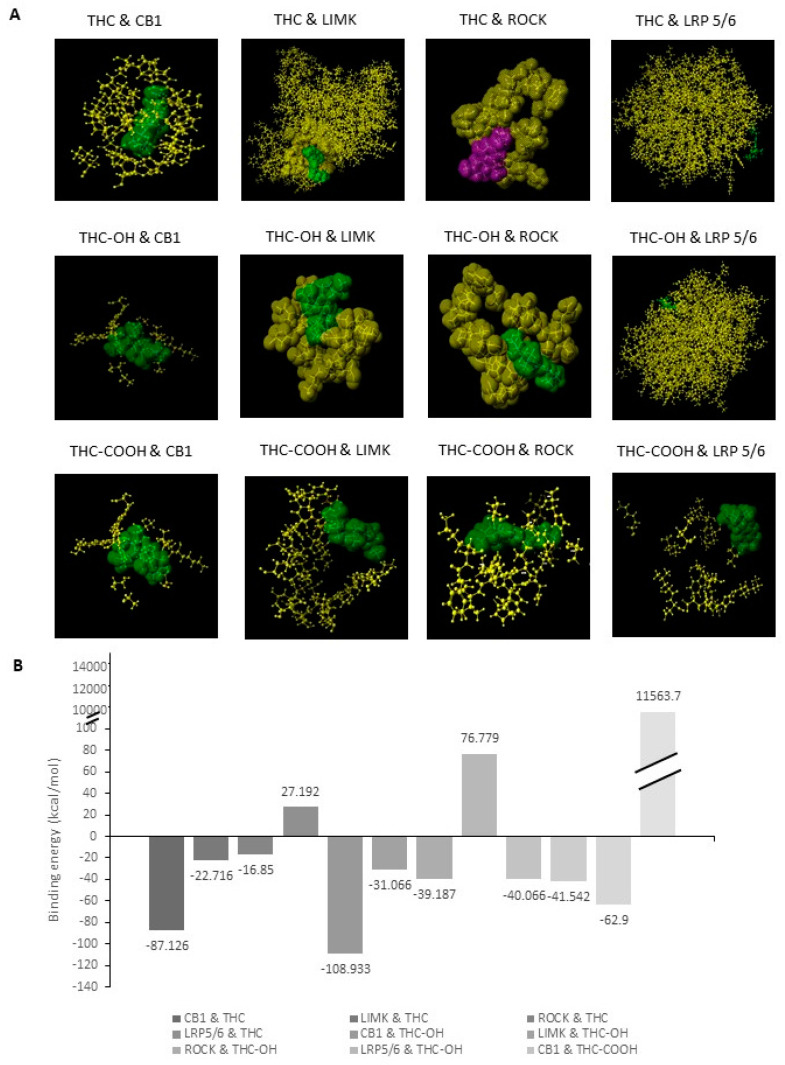
In-silico studies of THC and its metabolites THC-OH and THC-COOH modelled against key signalling regulators. (**A**) Interaction between ligands THC, THC-OH and THC-COOH with the ATP binding site known regulators; CB1, LIMK, ROCK, LRP5/6 and compared to CB1 as a positive control of a binding site that is permissive to cannabinoid activation. (**B**) Binding energies measured in kcal/mol generated by in-silico studies of THC and its metabolites THC-OH and THC-COOH modelled against key signalling regulators. (**A**,**B**) generated using the Scigress docking software.

Downstream kinases of the Rho-family GTPases have been shown to regulate actin severing via phosphorylation of cofilin. Rho-ROCK-LIMK have been shown to be a central signaling nexus in the modulation of cofilin and actin severing. To identify potential targets of cannabinoid induced motility we investigated the binding affinity of THC and its metabolites for the Rho-GTPases in comparison to the CB receptors (Figure 3A,B). In-silico modelling of THC against LIMK at the ATP binding pocket suggested a higher binding energy of −22.716 kcal/mol was required for permissive binding to take place when compared to THC-OH and THC-COOH, which required −31.066 kcal/mol and 41.542 kcal/mol, respectively. Similarly, modelling of THC against ROCK indicates that a higher binding energy of −16.85 kcal/mol is required for permissive binding to take place compared to THC-OH with −39.187 kcal/mol and THC-COOH with −41.542 kcal/mol. In-silico modelling suggests that THC-OH and THC-COOH have potential binding activity of Rho-ROCK-LIMK with potential for downstream upregulation of filamentous actin severing by ADF cofilin. By contrast, association of cannabinoids with the canonical Wnt receptor, LRP5/6, presented high binding energies suggesting non-permissive binding. Canonical Wnt signaling has previously been implicated in cytoskeletal rearrangement and increased matrix protein deposition.

### 2.4. Cannabinoids Upregulated Matrix Protein Expression in Cardiomyocytes

**Figure 4:**
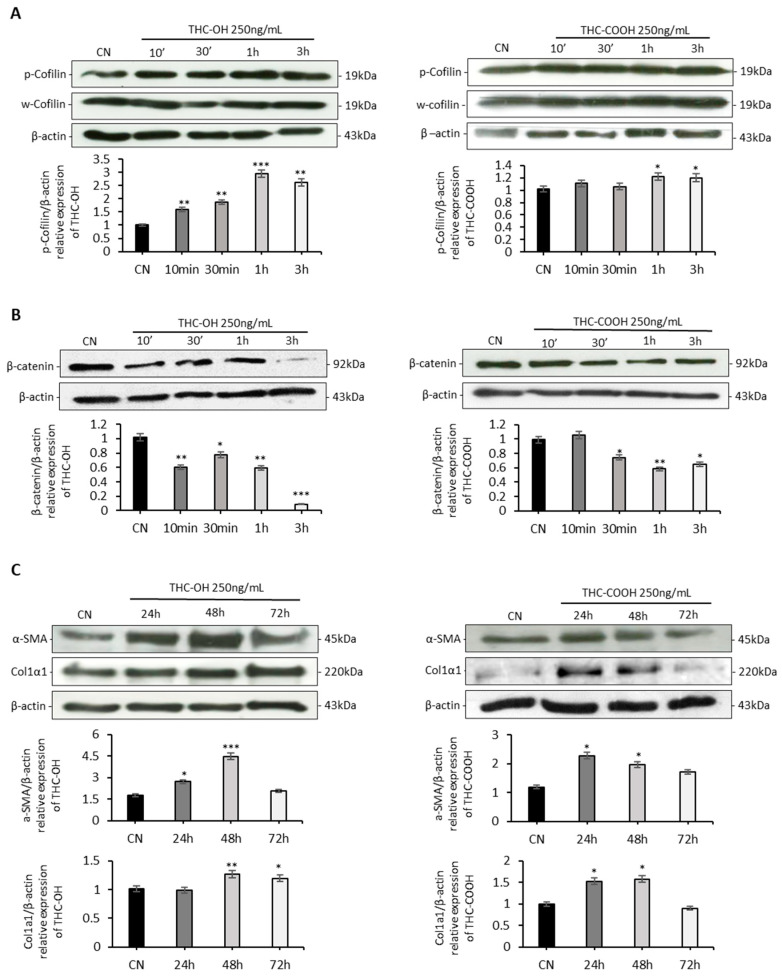
Cannabinoids differentially modulate ABP and ECM proteins. (**A**) Immunoblot analysis of Cofilin phosphorylation following THC-OH and THC-COOH exposure in H9c2 cardiomyocytes. (**B**) Time dependent protein detection of β-catenin following THC-OH and THC-COOH. (**C**) Western blot analysis of α-SMA and Col1α1 following THC-OH and THC-COOH. (**A**–**C**) Representative immunoblots of three independent experiments. Drug treatment concentration for both THC-OH and THC-COOH were 250 ng/mL. Densitometry indicating relative band expression for each blot measured using ImageJ software. Statistical comparison for treated vs. control was performed by a two-tailed Students unpaired *t*-test (* *p* < 0.05, ** *p* < 0.01 and *** *p* < 0.001) for individual time points.

In contrast to the results of the ligand binding studies, protein quantification experiments indicate that cannabinoids do not modulate cell migration via actin severing activity of cofilin. In agreement with previous ligand binding experiments cannabinoid metabolites also do not increase β-catenin expression, and these results are indicative of non-activation of the canonical Wnt signaling and RHO-ROCK Kinase modulation (Figure 4A,B).

ECM protein deposition is a key indicator of the pathogenesis of fibrogenesis, with numerous fibrotic conditions characterised by upregulation of key matrix proteins, such as collagen 1 and α-SMA (Figure 4C). H9c2 cardiomyocytes exposed to 250 ng/mL THC-OH and THC-COOH showed a statistically significant increase (*p* < 0.001) in α-SMA expression within 48 h before returning to basal levels within 72 h. Collagen 1 expression was significantly increased within 24–48-h of 250 ng/mL THC-OH (*p* < 0.005) and THC-COOH (*p* < 0.05) treatments. These findings suggest that cannabinoid metabolites increase the production of matrix proteins in H9c2 cells in vitro.

### 2.5. In-Vitro Analysis of Cannabinoid Metabolism in Cardiomyocytes

An LC-MS analysis technique was developed to identify the metabolic capacity of H9c2 cells in response to cannabinoid treatment. The analyte library consisted of three ion transitions for THC, THC-OH and THC-COOH, while the deuterated internal standards were identified using single ion transitions (Table 1). The analytical technique displayed a high degree of accuracy with quality control samples being detected within 92–97% of target concentration (Table 1). Our results suggest that following THC treatment, there is no metabolism or conversion of THC into THC-OH or THC-COOH after 24 h, furthermore treatments with THC-OH did not result in any metabolites for THC-COOH forming (Table 1). Our findings indicate that H9c2 cells do not possess the enzymatic capacity to metabolize THC into its primary metabolites. These results highlight a critical issue that should be considered with future in vitro cannabinoid assays, namely the lack of biotransformation of parent drugs into active and inactive metabolites which can elicit their own unique effects separately.

### 2.6. Cannabinoids Induce Alteration in the Microstructural of H9c2 Cells

**Figure 5:**
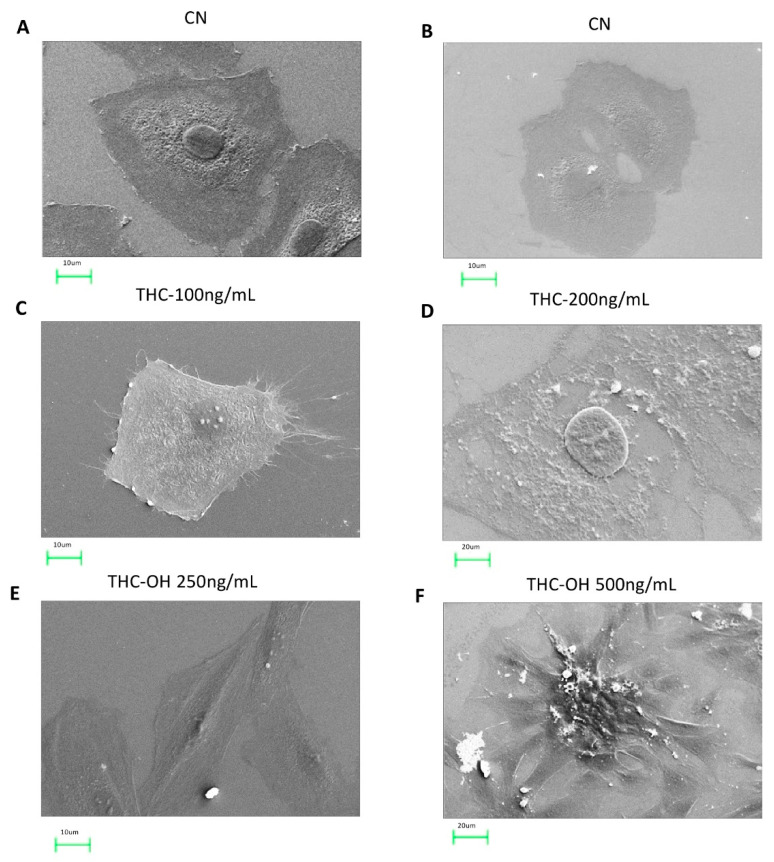

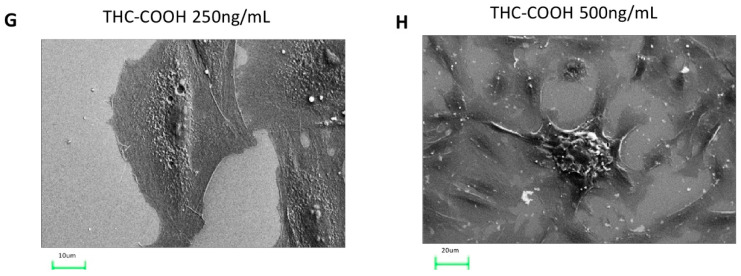
Dose dependent cannabinoid treatments result in morphological alterations of H9c2 cardiomyocytes. (**A**–**D**) In response to both low dose and high dose THC treatments, 100–200 ng/mL, normal H9c2 cardiomyocytes morphology was retained with intercellular connections and spindle-like spreading present at 24 h following culture. (**E**–**H**) Morphological alterations induced in H9c2 cardiomyocytes following exposure to 250 ng/mL THC-OH or THC-COOH. H9c2 cardiomyocytes indicated features of altered microstructural architecture, with membrane polarization, cell-substrate adhesion and retraction observed posterior to sites of membrane folding. Scale bar for each panel are presented below the respective image with numerical assignment indicating feature specific responses.

H9c2 cells were imaged using SEM to investigate if dose dependent cannabinoid treatments (THC 100/200 ng/mL THC-OH and THC-COOH 250/500 ng/mL) resulted in morphological alterations to cardiomyocytes. In response to THC treatment, there was no significant change in cell morphology compared to control (Figure 5A–D). Conversely, in response to low dose treatments with THC-OH and THC-COOH increased cell polarization occurred with pronounced stress fibre formation. Additionally, cells acquired a stellate shape concomitant with changes in cell adhesion; these morphological changes are consistent with early stages of increased cell motility (Figure 5E,G). High dose drug concentrations resulted in a deterioration of membrane integrity with perforations visible in response to THC-COOH and exacerbations in stress fibre formation in responses to high doses of THC-OH (Figure 5F,H). These results indicate that the level of polarization of cardiomyocytes is dose-dependent and more prominent in cells treated with THC-OH and THC-COOH.

### 2.7. Cannabinoids Modulate Regenerative Capacity of Polycelis nigra in a Dose Dependant Manner

**Figure 6:**
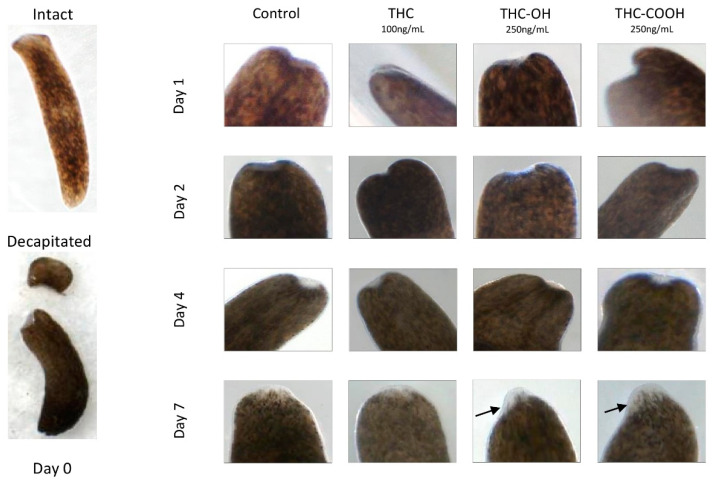
Low dose cannabinoids modulate regenerative capacity of *Polycelis nigra*. The effects of cannabinoids at low doses on the planarian flatworm regenerative capacity was assessed over 7 days. Decapitated planaria were treated with THC (100 ng/mL), THC-OH and THC-COOH (both 250 ng/mL) for one week after decapitation Arrows show increased regenerating areas.

**Figure 7:**
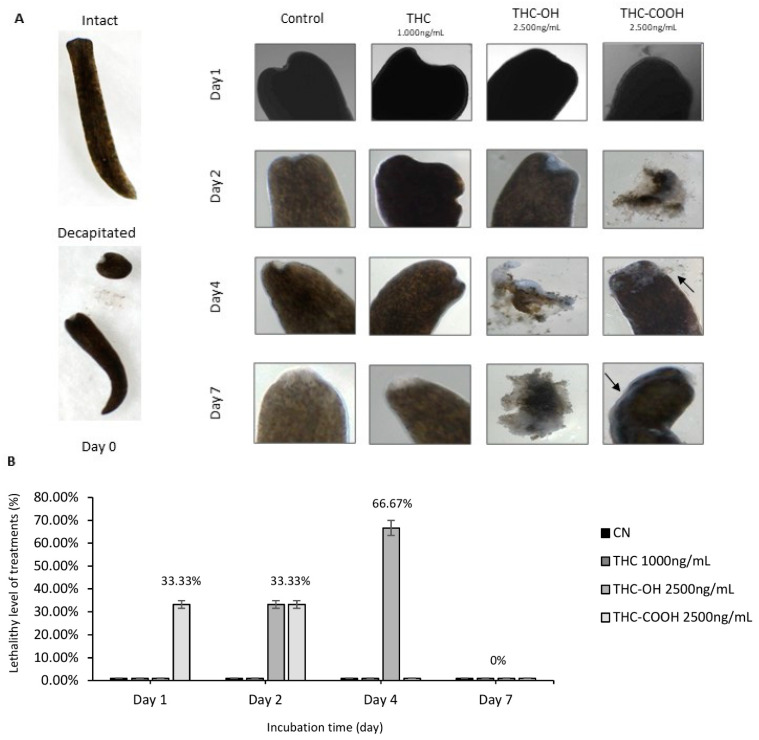
High dose cannabinoids treatments induce toxicity in *Polycelis nigra*. (**A**) High dose drug concentration treatment of THC (1000 ng/mL), and its metabolites (both 2500 ng/mL) in decapitated planaria observed for a week for regeneration and toxicity. (**B**) THC-OH and THC-COOH demonstrated toxicity in high dose, while THC-OH caused rapid disintegration and THC-COOH showed slower disintegration in which structural integrity was protected for a longer period of time. The arrows highlight the slower disintergration seen in THC-COOH treatments versus that of TH-OH.

Dose response studies using an in vivo regenerative model, the planarian flatworm species *Polycelis nigra* were undertaken to investigate alterations in the regenerative capacity of planaria following decapitation. Treatments with both low and high doses of THC (100 ng/mL and 1000 ng/mL) resulted in no overall change in structure or regeneration compared to control (Figure 6 and Figure 7A). However, increased cellular deposition was observed on the transection wound sites, suggestive of amplification of tissue deposition and rapid wound healing in response to 250 ng/mL doses of THC-OH and THC-COOH (Figure 6, Table 2). Conversely, increased THC-OH and THC-COOH (2500 ng/mL) concentrations resulted in a significant incidence of lethality amongst planaria with a decreased rate of regeneration of tissue and slow response to stimuli in surviving organisms (Figure 7A,B, Table 3). These results corroborate the findings of the in vitro H9c2 experiments wherein low doses of cannabinoid metabolites resulted in increased wound healing response coupled to upregulated ECM deposition, while high doses cannabinoid treatments resulted in cell toxicity and death.

## 3. Discussion

The current study investigated the roles of THC, THC-OH and THC-COOH in mediating phenotype changes in rat cardiomyocyte cells. The H9c2 cell line is effective in portraying human in vitro conditions for modulating cardiac specific markers and elucidating drug-induced toxic events [37]. A plethora of previous literature concentrated on the psychotropic and subsequent consequences of cannabis exposure on neuronal function [38,39,40]. While a number of recent studies have reported adverse cardiac events such as arrhythmia, acute coronary syndrome, and myocardial infarction in response to cannabis use, fewer studies have investigated the mechanism of THC and its metabolites on the cardiovascular environment [21,22,24]. To date, a mechanism mediating morphological changes in response to cannabinoids has not been proposed.

The present study reports dose dependant negative alterations in cardiac cell viability and morphology in response to THC-OH and THC-COOH treatments. By contrast, THC treatments mediated minimal alterations in cell structure or toxicity. SEM images identified stress fibre formation and cell elongation in response to 250 ng/mL THC-OH and THC-COOH treatments, indicative of cell polarisation and cytoskeletal alterations. Protein quantification by immunoblot analysis suggest increased phosphorylation of cofilin, a response associated with reduced actin severing and decreased cell motility [41]. These results suggest that actin disassembly via cofilin activation is unlikely to be the mechanism responsible for increased cell migration in response to cannabinoid treatments. Our investigations also indicate that in response to treatments with THC-OH and THC-COOH, H9c2 cardiomyocytes exhibit a downregulation in the expression of β-catenin. By contrast activation of LRP5/6, and subsequent canonical Wnt signalling, is characterised by increased β-catenin concentrations and subsequent translocation to the nucleus and followed by TCF-LEF transcription [42]. These results are further supported by the in-silico docking studies of THC-OH and THC-COOH which suggest an energetically unfavourable affinity for the LRP5/6 ATP binding pocket. The canonical Wnt/β-catenin signalling pathway is an important regulator of cell adhesion, embryonic development and adult homeostasis and previously been linked with numerous fibrotic phenotypes. However, our findings do not identify this signalling nexus as a mediator of increased cell motility and morphological changes in response to cannabinoids [43,44].

By contrast, the current study identified an upregulation in phosphorylation of AKT and ERK1/2 in response to 250 ng/mL treatment with THC-OH and THC-COOH, respectively. The PI3K/AKT and ERK/MAPK signalling pathways are important regulators of cell proliferation, apoptosis, and motility. Additionally, increased cell proliferation in response to THC-OH and THC-COOH, concomitant with modulation of PI3K and ERK/MAPK suggest that these physiological changes may be an AKT microtubule driven event. PI3K/AKT and ERK/MAPK pathways have previously been identified as targets for cancer therapy as dysregulation of this signalling nexus leads to aberrant signalling activation and tumorigenesis [45,46]. Conversely, several studies suggest that upregulation of AKT in cardiac cells during ischaemia and reperfusion has positive implications. The pathophysiology of reperfusion injury is characterised by the restoration of blood flow to previously ischaemic tissue [47]. Ong and colleagues [48], investigated genetic and/or pharmacological activation of AKT in the HL-1 cardiac muscle cell line and concluded that acute activation of AKT presents cardioprotective qualities against ischaemic reperfusion injury through mitochondrial modulation, with other experimental studies also confirming this finding [49,50,51]. Moreover, it is important to highlight that research by Ong et al. [52] investigated the effect of acute activation of AKT as a cardioprotective measure whilst chronic AKT activation has been reported to cause cardiac hypertrophy. Similarly, ERK1/2 overexpression is implicated in several phenotypic forms of cardiac hypertrophy and progression to heart failure [53]. Cardiac hypertrophy can be reversible following clinical and dietary intervention however pathological hypertrophy of the heart caused by chronic stress exerted on the cardiac muscle through hypertension may increase ventricular wall dimension and be accompanied by fibrosis [54]. Uncontrolled and chronic upregulation of AKT as a result of continuous cannabis exposure is sufficient to induce phenotypic expression changes ranging from cardiac hypertrophy with preserved systolic function to cardiac dilation and sudden death [52]. Similarly, ERK1/2 overexpression is implicated in several phenotypic forms of cardiac hypertrophy and progression to heart failure [53].

Numerous studies have indicated that following smoking of a single preparations of cannabis, THC blood concentrations of 100–150 ng/mL were achieved within 10 min of inhalation, after which THC concentrations decline rapidly [55]. Due to the slow elimination of THC-COOH, regular users of the drug can still present concentrations of up to 50 ng/mL in whole blood following 7 days of abstinence, this was also coupled with for residual neurocognitive impairment observed in chronic cannabis users [56]. Research studies in which cannabinoid metabolites were measured in patients following a single 6.8% THC (*wt*/*wt*) cannabis detected concentrations ranging from 50–460 ng/mL in plasma and whole blood [57]. The metabolism of THC predominantly occurs as a hepatic level event, however, extrahepatic metabolism also occurs in tissues of the brain, heart and lungs which exhibit the CYP450 enzymes [58]. Oxidative metabolism of THC via a microsomal reaction through the CYP450 enzymatic pathway yields the active metabolite, THC-OH, and inactive metabolite, THC-COOH [59]. Our results demonstrate that in vitro H9c2 cardiomyocytes models do not provide the required environment for THC metabolism. Cannabinoid concentrations in cell media were quantified using LC-MS analysis and demonstrate no formation of either THC-OH or THC-COOH even 24 h after THC administration. This is significant as cell-based assays investigating THC treatments with no oxidative or CYP450 enzymes present are significantly limited in their scope to assess the effects of THC metabolism and it subsequent break down products on organ function and cell viability. While previous studies have suggested an anti-inflammatory and tissue protective effect for THC, this may not be replicated in vivo settings or cases of chronic recreational use where the half-life of THC-COOH is significantly longer and may counteract the beneficial effects of THC.

The deleterious effects of THC-OH and THC-COOH on cell integrity and structure was further demonstrated in SEM imaging studies where high dose treatments of both metabolites resulted in pronounced cell toxicity, characterised by perforations of the cell membrane, deterioration in cytoskeletal structure and irregular nuclei. This cytotoxic effect was further investigated utilising an animal model, *Polycelis nigra* planaria, to observe if cannabinoids altered regeneration or wound healing dynamics. The Planaria species was chosen for this study due to their notable tissue regenerative properties [60], making them an ideal model to investigate dysregulation in regeneration and dose response toxicity [61,62,63,64]. Other research groups have utilised this animal model to investigate mechanism of drug abuse and dysregulation of neuronal function [20,63,65]. The current study demonstrated that in response to dose-dependent treatments of THC-OH and THC-COOH, the regeneration rate of amputated *Polycelis nigra* is differentially modulated. Following drug exposure for 7 days, during which regeneration dynamics were quantified, low dose of THC metabolites, THC-OH and THC-COOH (250 ng/mL) accelerated the blastema formation. This resulted in increased tissue deposition volume and area at the peripheral of wound healing sites, with accelerated wound healing dynamics and increased tissue deposition compared to control treatments. At tenfold higher concentrations of THC-OH and THC-COOH (2500 ng/mL), severe toxicity and planaria disintegration occurred, suggesting that low doses lead to rapid changes in cellular dynamics consistent with dysregulated wound healing while higher doses are cytotoxic to the organ and cell function. As with SEM and scratch wound assays, THC had no significant effect on cytotoxicity or regeneration even at increased concentrations of 1000 ng/mL.

This study has identified two major cannabinoid metabolites, THC-OH and THC-COOH as mediators of increased cell migration, matrix protein deposition, cytoskeletal rearrangement, and dose dependant cell toxicity. Characteristics consistent with cell necrosis and fibrotic processes that govern cardiac dysregulations including cannabis-induced ischemic stroke and myocardial infarction following cannabis use [35]. Moreover, these results elucidate the critical role that the less pharmacologically active metabolites such as THC-COOH, play in opposing protective functions of cannabinoid receptor agonism. Future studies can identify increased cardiac toxicity and subsequent cell injury as a precursor to enhanced activation of cardiac fibroblasts following repeated dosages of cannabinoid metabolites. Long term, these effects are likely to result in significant alterations to the morphology of the cardiac architecture. Our results suggest that cannabinoid metabolites have the capacity to increase cell toxicity and matrix protein deposition in the cardiac milieu, these dual events are frequently early hallmarks of cardiac hypertrophy and fibrosis as endogenous myocyte tissue is replaced with non-contractile ECM protein.

Numerous studies have reported myocardial dysregulation following cannabis abuse, with myocardial infarction induced by vascular spasm and vasoconstriction being proposed as a mechanism of cannabis induced cardiovascular complications [7,36,66]. Chronic cannabis use has been linked to hepatotoxicity and structural alterations of the liver, with elevated liver markers consistent with hepatic sclerosis [67]. Conversely, the cardiac structure does not possess the regenerative capacity of the liver, if metabolites of cannabis induce severe structural alterations that result in organ hypertrophy, increased matrix protein deposition followed by reduced contractility, and at chronic repeated concentrations, cell death; these effects are likely to result in an irreversible decline of cardiac function.

Even in cases that do not progress to full fibrosis, cardiac hypertrophy remains a significant risk due to changes in the heart structure and replacement of endogenous tissue with enlarged ECM proteins. Regardless, either condition is associated with poor prognosis and declining future cardiac function due to loss of contractile capacity. Future studies investigating the possible role of cannabinoid metabolites in the progressive decline of cardiac function can contribute to greater understanding of how cannabis use can lead to increased mortality and morbidity.

## 4. Materials and Methods

### 4.1. Chemical and Reagents

The following chemicals and reagents were utilized in the experiments explained below. Δ9-THC 1.0 mg/mL, 11-Hydroxy-Δ9-THC 100 µg/mL and 11-nor-9-Carboxy-Δ9-THC 100 µg/mL in methanol (Cerilliant, Merck, Round Rock, TX, USA) were used for all treatments and diluted in either DMEM, Dulbecco’s phosphate-buffered saline (DPBS) (Sigma-Aldrich, St Louis, MO, USA), or planaria water. Dimethethyl sulfoxide (DMSO) (Sigma-Aldrich, St Louis, MO, USA) was used to prepare freezing medium during cryopreservation procedure. The BrdU incorporation assay (Merck Millipore, Kenilworth, NJ, USA) kit was used for cell proliferation analysis. Radioimmunoprecipitation assay (RIPA) buffer (Sigma-Aldrich, St. Louis, MO, USA) supplemented with protease inhibitor cocktail (1:100) (Abcam, Cambridge, UK), phosphatase inhibitor cocktail (1:100) (Sigma-Aldrich, St Louis, MO, USA) and 1 mM phenylmethanesulfonyl fluoride solution (Sigma-Aldrich, St. Louis, MO, USA) was used to perform the cell lysing procedure. In order to quantify protein in each cell lysate, a Bradford reagent (Sigma-Aldrich, St Louis, MO, USA) was used, and the protein samples were prepared by using NuPAGE LDS Sample Buffer 4X (Thermo Fisher Scientific, Waltham, MA, USA) containing 2% (*w*/*v*) dithiothreitol (DTT) (Thermo Fisher Scientific, Waltham, MA, USA) for immunoblotting assays. Bovine-serum albumin (BSA) (Sigma-Aldrich, St Louis, MO, USA) was used to prepare BSA standards for the Bradford quantification assay. The primary antibodies used for immunoblotting assays are detailed in Section 4.2.4. Organic mobile phase HPLC grade Acetonitrile and aqueous HPLC grade water (both VWR Chemicals, Radnor, PA, USA) were used for LC-MS analysis, both mobile phases were supplemented with 0.1% formic acid (Sigma Aldrich, St Louis, MO, USA) and only into organic solution 0.01g/L of ammonium acetate was added. Δ9-THC-D,3, 1.0 µg/mL and 11-nor-9-Carboxy-Δ9-THC-D3, 1.0 µg/mL were used as reference standards (Cerilliant, Gillingham, UK). Scanning Electron Microscopy (SEM) fixation was carried out using 2.5% (*v*/*v*) glutaraldehyde solution (Sigma-Aldrich, St Louis, MO, USA) and 1% (*w*/*v*) osmium tetraoxide (OsO4) (Agar Scientific Stansted, UK). Dehydration was performed using graded ethanol solutions (VWR Chemicals, Radnor, PA, USA) and hexamethyledisilizane (Sigma-Aldrich, St Louis, MO, USA). For the toxicity and regeneration experiment, planaria (*Polycelis nigra*) and planaria water was used.)

### 4.2. Experimental Methods

#### 4.2.1. Scratch Wound Assay

H9c2 embryonic cardiomyocyte (Lonza, Slough, UK) cell sub-clones, derived from the parental clone cell line embryonic BD1X rat heart tissue was used in this study. The H9c2 cardiomyocyte cell line has widely been utilized for cardiotoxicity studies for the analysis of morphological characteristics, as changes in cellular activity resemble immature embryonic cardiomyocytes with many signalling pathways conserved for their differentiation into mature myocardial cells. Furthermore, the H9c2 cardiomyocyte cell line has commonly been used in various studies relating to drug toxicity pathways (American Type Culture Collection (ATCC)) and to this end provided a viable in vitro model [63]. Cells were either subcultured into 75 cm^2^ sterile flasks at the required culture ratio of either 1:5 or 1:10 or seeded at adequate densities into 6-well or 96- well tissue culture plates. Initial cell starting passage was between 4 and 6 with all experiments performed between passages 8–12.

To analyse the migratory response of H9c2 cardiomyocytes following cannabinoid exposure on wound repair, cells were allowed to reach a 70–80% confluency in clear sterile 6-well culture plates (Corning Costar Incorporated, Cambridge, MA, USA). After a monolayer was formed, a stimulated scratch wound was applied using a 200 µL sterilised plastic pipette. High glucose (4500 mg/L) DMEM (Sigma Aldrich, St Louis, MO, USA) supplemented with 10% Foetal Bovine Serum (FBS), 1% (100 U/mL) penicillin, (100 g/mL) streptomycin, L-glutamine, sodium bicarbonate and 100 mM sodium pyruvate were used to culture H9c2 cardiomyocytes. The cells grown in complete cell growth medium alone served as a control group. The cells were treated with 100 ng/mL of THC, 250 ng/mL of THC-OH and 250 ng/mL of THC-COOH supplemented with complete medium and incubated for 24 h at 37 °C in a 5% CO_2_ incubator. Images were captured with AMG Evos FL™ inverted microscope at 0 h and after 24 h. From three selected areas on the wound, measurements were taken using the ImageJ Image Processing and Analysis software™, (Research Services Branch, National Institute of Mental Health, Bethesda, MD, USA) and percentage of wound closure was determined. Measurements were statistically analysed from three independent experiments using the IBM SPSS Statistics software package (Version 26.0.0.0, 64-bit edition, Armonk, NY, USA). Statistical analysis was performed by a two-tailed Students unpaired *t*-test, * *p* < 0.05 for treated vs. control.

#### 4.2.2. Cell Proliferation Assay

The H9c2 cell proliferation assay was measured in response to 100 ng/mL of THC, 250 ng/mL of THC-OH and 250 ng/mL of THC-COOH for 24h, by the colorimetric read-out of BrdU incorporation assay (Merk Millipore, Watford, UK). Untreated cells served as the control group. H9c2 cardiomyocytes were cultured in sterile 96- well tissue plate and incubated with BrdU label diluted in complete DMEM (1:2000). Following removal of the BrdU label, H9c2 cardiomyocytes were fixed and denatured with BrdU Fixative/Denaturing solution, then cells were labelled with anti-BrdU conjugated antibody (1:100). BrdU labelled H9c2 cells were then washed with 1× diluted washing buffer and labelled with reconstituted anti-BrdU-peroxidase conjugate antibody (1:1000). Following washing of peroxidase-labelled cells and exposed with the chromogenic peroxidase substrate, tetra-methylbenzidine (TMB), stop solution was added prior to take the plate for absorbance measurement using a Tecan Infinite M200 PRO spectrophotometer at OD 450 nm and 540 nm. Statistical comparison was performed by a two-tailed Students unpaired *t*-test, * *p* < 0.05 for treated vs. control from five independent experiments.

#### 4.2.3. In-Silico Bioinformatic Docking Studies

The Scigress Fujitsu™ software Version 3.4. (Tokoyo, Japan) was used in order to conduct molecular modelling and drug ligand-docking analyse for the interaction of ligands: THC, THC-OH and THC-COOH with the ATP binding site in known key signalling regulators: CB1, LIMK, ROCK and LRP 5/6. Relevant molecular structures of THC, THC-OH and THC-COOH were programmed into the software before molecular modelling in three-dimensional (3D) conformer SDF format. Peptide sequences were imported in FASTA format into the SWISS-MODEL protein structure homology-modelling online server to generate 3D protein structural models derived from X-ray diffraction and NMR studies. To measure the protein domain specific sequences, structural coverage levels were utilized. Generated structural models were imported as a PDB format into the Scigress Fujitsu™ software Version 3.4 (Tokoyo, Japan) to manage according to specific domains of interest. Binding energy graphs in kilocalorie/mole for THC, THC-OH and THC-COOH with each key modulators were produced for specified docking sites of interest.

#### 4.2.4. Western Blotting

H9c2 cardiomyocytes were cultured in clear sterile 6-well culture plates (Corning Costar^®^) with complete DMEM (Sigma-Aldrich, St Louis, MO, USA). After drug exposure of 100 ng/mL of THC, 250 ng/mL of THC-OH and 250 ng/mL of THC-COOH at different time points (10 min, 30 min, 1 h, 3 h, 24 h, 48 h, and 72 h), cells were lysed using RIPA buffer supplemented with protease inhibitor cocktail (1:100), phosphatase inhibitor cocktail (1:100) and 1 mM phenylmethanesulfonyl fluoride solution. For the Bradford assay, Bradford reagent and BSA standards (both Sigma-Aldrich, St Louis, MO, USA) were used to quantify protein in each lysate. The protein samples were prepared with using NuPAGE LDS Sample Buffer 4X containing 2% (*w*/*v*) dithiothreitol DTT (Thermo Fisher Scientific, Waltham, MA, USA) for Western blotting assays. Total proteins were separated using 6%, 8%, 10% or 12% SDS–PAGE depending on the molecular weight of the protein of interest, transferred to a nitrocellulose membrane using semi-dry electrotransfer and probed with the following antibodies derived from rabbit except AKT after blocking with 5% BSA or 5% non-fat dry milk solution: ERK1/2, Cofilin/P-Cofilin, β-catenin, AKT/P-AKT (mouse), α-SMA, Col1a1 and β-actin (rabbit). The secondary antibodies were horseradish peroxidase linked as either anti mouse or anti rabbit. All antibodies were purchased from Cell Signaling Technology. The primary antibodies were used at dilution of 1:1000 and 1:10,000 for the loading control (β-actin). The secondary antibodies were used at dilutions of 1:2000–5000 depending on the expression level of the primary antibody. Densitometry analysis on the Western blot bands was performed using the ImageJ Image Processing and Analysis software™ and the data was normalised to total protein levels.

#### 4.2.5. LC-MS Analysis

H9c2 cardiomyocytes were treated with 100 ng/mL of THC, 250 ng/mL of THC-OH and 250 ng/mL of THC-COOH and cell media was then subtracted at specific timepoints between 10 min and 24 h. The samples were spiked with deuterated internal standards for THC (THC-D3) and THC-COOH (THC-COOH-D3). One mL of cell media was added for each sample, calibrators, blanks, and quality control (QC) standards in conical centrifuge tubes (Fisher Scientific). Following the drug standards addition, samples were treated with chilled acetonitrile and centrifuged for 5 min at 3000 RCF. Supernatant was removed and treated with 2 M pH 4 acetate buffer, samples passed through an Strata C18-E (55 um, 70 A) 200 mg/3mL cartridges (Phenomenex, Macclesfield, UK) for extraction. Samples were eluted in a 70:30 hexane ethyl acetate mix prior to evaporation under nitrogen flow at 40 °C. Samples were then reconstituted in 160 µL of 50/50 of mobile phase A (H2O 0.1% formic acid) and mobile phase B (acetonitrile 0.1% formic acid). Drug samples were analysed using a LCMS/MS Agilent 6430 coupled with a Agilent 1260 UHPLC system. Chromatographic separation was achieved using an Agilent Infinity Lab Poroshell 120 EC-C18, 2.1 × 75 mm, 2.7 µm, narrow bore LC column. Mobile phases, were combination of aqueous and organic solutions, aqueous mobile phase consisted of water and 0.1% formic acid. Organic mobile phase was comprised of LC-MS grade acetonitrile and 0.1% formic acid. Further information on LC-MS analysis can be found in Figure S1.

#### 4.2.6. Scanning Electron Microscopy

Scanning Electron Microscopy (SEM) was used to visualise detailed morphological changes on H9c2 cardiomyocyte cells in response to THC at concentrations of 100 ng/mL and 200 ng/mL, THC-OH at concentrations of 250 ng/mL and 500 ng/mL, and THC-COOH at concentrations of 250 ng/mL of THC-COOH 500 ng/mL. Cells were cultured on sterile coverslips in 6-well plates (Corning Costar Cambridge, MA, USA), then primarily fixed using 2.5% (*v*/*v*) Glutaraldehyde solution diluted in 0.1M DPBS. After the primary fixation, H9c2 cells were washed in DPBS and the secondary fixation containing 1% (*w*/*v*) Osmium Tetraoxide (OsO4) was applied. Following the secondary fixation, H9c2 cardiomyocytes were dehydrated through graded ethanol concentration series (50, 70, 80, 90, 95 and 100% (*v*/*v*), respectively), then immersed in Hexamethyledisilizane and allowed to air dry in a fume cupboard. Dried coverslips mounted onto glass slides with double-sided conductive tape and exposed to gold palladium alloy sputter coating (Polaron Range SC7640). Thereafter, the coated specimens visualized using the Zeiss™ EV050 SEM to capture polarised H9c2 cells.

#### 4.2.7. Planaria Studies

To determine the effect of low and high dose of THC, THC-OH and THC-COOH exposure on toxicity and regeneration, planaria (*Polycelis nigra*) was implemented as an animal model due to possessing a central nervous system and regenerative capacity. In a 6-well plate, 100 ng/mL of THC, 250 ng/mL of THC-OH, and 250 ng/mL of THC-COOH, respectively were diluted in planaria water (0.5 g Ocean Salts/1 L ddH_2_O). Planaria water not spiked with cannabinoids served as a control. A dose-dependent experiment required the preparation of planaria water spiked with 10× higher concentrations: 1000 ng/mL of THC, 2500 ng/mL of THC-OH and 2500 ng/mL of THC-COOH. Following the observation of intact planaria under the dissecting microscope (Motic, Barcelona, Spain), planaria were decapitated with a sterile disposable scalpel (Swann-Morton Ltd, Sheffield, UK) and then placed in cannabinoid or control conditions. For each studied cannabinoid concentration, each well contained three decapitated planaria with retained anterior (head) and posterior (body) parts. Regenerating planaria were maintained at ≈21 °C in dark conditions. Cannabinoids in planaria water and control planaria water were replenished every 2–3 days to maintain compound activity and the wound healing of each planaria was observed for 7 days under a Motic dissecting microscope.

## Figures and Tables

**Table 1:** H9c2 cardiomyocytes do not metabolize cannabinoids in vitro. (**A**) Ion transitions and mass spectrometry parameters for the LC-MS quantification method of cannabinoids (**B**) In all analysis quality control samples (QC) of extracted spiked media were run alongside samples. These QC demonstrated a calculated concentration accuracy of 90–100%. (**C**) THC treatments of 100 ng/mL resulted in a media concentration of 33 ng/mL after 10 mins of collection, with an approximate 30% decrease over 24 h in THC concentration.

(A)							
Analyte Name	Precursor ion (Amu)	Retention Time (min)	Retention Time Window (min)	Fragmentor (V)	Product Ions (amu)	Collision Energy (V)	Dwell Time (ms)
THC	315.2	14.4	1.19	84	193.2 123.0 259.2	21 32 18	78.64 78.64 78.64
THC-OH	331.2	8.8	1.01	92	313.2 201.0 193.2	20 26 15	77.48 77.48 77.48
THC-COOH	345.2	9.2	0.94	96	327.2 299.2 193.1	13 17 25	35.63 35.63 35.63
**(B)**							
**QC**		**Expected Concentration (ng/mL)**		**Calculated Concentration (ng/mL)**		**Accuracy (%)**	
THC		40.00		39.7692		99.42	
THC-OH		100.00		97.3173		97.32	
THC-COOH		100.00		92.9326		92.93	
**(C)**							
**Data File**		**THC** **Conc. (ng/mL)**		**THC-OH** **Conc. (ng/mL)**		**THC-COOH** **Conc. (ng/mL)**	
THC Control		0.0000		ND		1.1767	
THC 10 min		33.8205		ND		1.1408	
THC 30 min		33.8275		ND		1.1094	
THC 1 H		37.7523		ND		1.1627	
THC 3 H		38.1000		ND		1.1176	
THC 24 h		20.8132		ND		1.1047	
THC-OH control		ND		1.5706		1.1057	
THC-OH 10 min		ND		143.4358		1.1159	
Hydroxy-THC 30 minl		ND		129.2503		1.1364	
THC-OH 1 h		ND		121.2555		1.1277	
THC-OH 3 h		ND		106.0970		1.1277	
THC-OH 24 h		ND		91.2759		1.1374	

**Table 2:** Treatment with THC (100 ng/mL) resulted in no overall change in structure or regeneration compared to control. Conversely, increased cellular deposition was observed on the transection wound sites in response to 250 ng/mL doses of THC-OH and THC-COOH (shown in Figure 6). N = 3 per condition.

Treatment Condition	Disintegration	Wound Healing at Day 1	Obvious Blastema at Day 4	Presence of Regenerated Tissue at Day 7	Regeneration Rate at Day 7 Compared to Control
**Control**	No	Yes	Yes	Yes	--
**THC 100 ng/mL**	No	Yes	Yes	Yes	No difference to control
**THC-OH 250 ng/mL**	No	Yes	Yes	Yes	Increased thickness of regenerated tissues
**THC-COOH 250 ng/mL**	No	Yes	Yes	Yes	Increased thickness of regenerated tissues

**Table 3:** Detailed data for the effect of high concentration treated planaria for a week.

Treatment Condition	Wound Healing at Day 1	Disintegration	Obvious Blastema at:	Presence of Regenerated Tissue at:	Regeneration Rate at Day 7 Compared to Control
**Control**	Yes	No	Day 4	Day 7	---
**THC 1000 ng/mL**	Yes	No	Day 7	Day 7	No difference to control
**THC-OH 2500 ng/mL**	Yes	Disintegrated completely between days 2–4	Day 1	Day 2	N/A; completely disintegrated
**THC-COOH 2500 ng/mL**	Yes	Disintegrated completely at day 1 (n = 1) and day 2 (n = 1)	Day 1	Day 4	N/A; completely disintegrated except 1 individual with slow response to stimuli and little regeneration

## Data Availability

Not applicable.

## Supplementary Materials

The following supporting information can be downloaded at: https://www.mdpi.com/article/10.3390/ijms23031401/s1.

## Abbreviations

ABP	Actin binding protein
ACS	Acute Coronary Syndrome
AKT	Protein Kinase B
αSMA	Alpha Smooth Muscle Actin
ATP	Adenosine triphosphate
BrdU	Bromo-2′-deoxy-uridine
CB	Cannabinoid
DPBS	Dulbecco’s Phosphate Buffered-Saline
DMEM	Dulbecco’s modified eagle medium
ECM	Extracellular matrix
ERK	Extracellular regulated kinase
FASTA	Format DNA and Protein Sequence Alignment
FBS	Fetal bovine serum
FN	Fibronectin
LC-MS	Liquid Chromatography Mass Spectrometry
LIMK	LIM-kinase
MAPK	Mitogen-Activated Protein Kinase
PI3K	Phosphatidylinositol 3-kinases
RIPA	Radioimmunoprecipitation assay
ROCK	Rho- associated kinase
SEM	Scanning Electron Microscopy
THC	Δ9-tetrahydrocannabinol
THC-COOH	11-nor-9-carboxy-Δ⁹-tetrahydrocannabinol
THC-OH	11-Hydroxy Δ9-tetrahydrocannabinol

## References

[1] Knuth M., Temme O., Daldrup T., Pawlik E. (2018). Analysis of cocaine adulterants in human brain in cases of drug-related death. Forensic Sci. Int..

[2] Mittleman M.A., Mintzer D., Maclure M., Tofler G.H., Sherwood J.B., Muller J.E. (1999). Triggering of Myocardial Infarction by Cocaine. Circulation.

[3] Egred M., Viswanathan G., Davis G.K. (2005). Myocardial infarction in young adults. Postgrad. Med. J..

[4] Maceira A.M., Ripoll C., Cosin-Sales J., Igual B., Gavilan M., Salazar J., Belloch V., Pennell D.J. (2014). Long term effects of cocaine on the heart assessed by cardiovascular magnetic resonance at 3T. J. Cardiovasc. Magn. Reson..

[5] Wang Z.-J., Martin J.A., Gancarz A.M., Adank D.N., Sim F.J., Dietz D.M. (2017). Activin A is increased in the nucleus accumbens following a cocaine binge. Sci. Rep..

[6] Lee T.-M., Lin S.-Z., Chang N.-C. (2014). Membrane ERα attenuates myocardial fibrosis via RhoA/ROCK-mediated actin remodeling in ovariectomized female infarcted rats. J. Mol. Med..

[7] Pereira J., Saez C.G., Pallavicini J., Pereira-Flores K., Mendoza C., Hernández R., Novoa U., Ocaranza M.P., Massardo T., Mezzano D. (2012). Cocaine-Induced Endothelial Dysfunction: Role of RhoA/Rho Kinase Pathway Activation. Blood.

[8] Pradhan L., Mondal D., Chandra S., Ali M., Agrawal K.C. (2008). Molecular Analysis of Cocaine-Induced Endothelial Dysfunction: Role of Endothelin-1 and Nitric Oxide. Cardiovasc. Toxicol..

[9] Zhang J., Fan G., Zhao H., Wang Z., Li F., Zhang P., Zhang J., Wang X., Wang W. (2017). Targeted inhibition of Focal Adhesion Kinase Attenuates Cardiac Fibrosis and Preserves Heart Function in Adverse Cardiac Remodeling. Sci. Rep..

[10] Martins M.J., Bravo R.R., Enea M., Carmo H., Carvalho F., Bastos M.D.L., Dinis-Oliveira R.J., Da Silva D.D. (2018). Ethanol addictively enhances the in vitro cardiotoxicity of cocaine through oxidative damage, energetic deregulation, and apoptosis. Arch. Toxicol..

[11] Yang D., Liu W., Ma L., Wang Y., Ma J., Jiang M., Deng X., Huang F., Yang T., Chen M. (2017). Profilin-1 contributes to cardiac injury induced by advanced glycation end-products in rats. Mol. Med. Rep..

[12] Graziani M., Sarti P., Arese M., Magnifico M.C., Badiani A., Saso L. (2017). Cardiovascular Mitochondrial Dysfunction Induced by Cocaine: Biomarkers and Possible Beneficial Effects of Modulators of Oxidative Stress. Oxidative Med. Cell. Longev..

[13] Manninger M., Perl S., Brussee H., Toth G.G. (2018). Sniff of coke breaks the heart: Cocaine-induced coronary vasospasm aggravated by therapeutic hypothermia and vasopressors after aborted sudden cardiac death: A case report. Eur. Hear. J. Case Rep..

[14] Calipari E., Godino A., Salery M., Damez-Werno D.M., Cahill M., Werner C.T., Gancarz A.M., Peck E.G., Jlayer Z., Rabkin J. (2019). Synaptic Microtubule-Associated Protein EB3 and SRC Phosphorylation Mediate Structural and Behavioral Adaptations During Withdrawal From Cocaine Self-Administration. J. Neurosci..

[15] Jones C.M., Baldwin G.T., Compton W.M. (2017). Recent Increases in Cocaine-Related Overdose Deaths and the Role of Opioids. Am. J. Public Health.

[16] Wu S.-N., Chang H.-D., Sung R.J. (2006). Cocaine-Induced Inhibition of ATP-Sensitive K+ Channels in Rat Ventricular Myocytes and in Heart-Derived H9c2 Cells. Basic Clin. Pharmacol. Toxicol..

[17] Fan L., Sawbridge D., George V., Teng L., Bailey A., Kitchen I., Li J.-M. (2008). Chronic Cocaine-Induced Cardiac Oxidative Stress and Mitogen-Activated Protein Kinase Activation: The Role of Nox2 Oxidase. J. Pharmacol. Exp. Ther..

[18] Lattanzio F.A., Tiangco D., Osgood C., Beebe S., Kerry J., Hargrave B.Y. (2005). Cocaine Increases Intracellular Calcium and Reactive Oxygen Species, Depolarizes Mitochondria, and Activates Genes Associated With Heart Failure and Remodeling. Cardiovasc. Toxicol..

[19] Havakuk O., Rezkalla S.H., Kloner R.A. (2017). The Cardiovascular Effects of Cocaine. J. Am. Coll. Cardiol..

[20] Moreira F.P., Medeiros J.R.C., Lhullier A.C., Souza L.D.D.M., Jansen K., Portela L.V., Lara D.R., da Silva R.A., Wiener C.D., Oses J.P. (2016). Cocaine abuse and effects in the serum levels of cytokines IL-6 and IL-10. Drug Alcohol Depend..

[21] Badisa R.B., Wi S., Jones Z., Mazzio E., Zhou Y., Rosenberg J.T., Latinwo L.M., Grant S.C., Goodman C.B. (2018). Cellular and molecular responses to acute cocaine treatment in neuronal-like N2a cells: Potential mechanism for its resistance in cell death. Cell Death Discov..

[22] Chandra R., Engeln M., Schiefer C., Patton M., Martin J.A., Werner C., Riggs L.M., Francis T.C., McGlincy M., Evans B. (2017). Drp1 Mitochondrial Fission in D1 Neurons Mediates Behavioral and Cellular Plasticity during Early Cocaine Abstinence. Neuron.

[23] Werner C.T., Mitra S., Auerbach B.D., Wang Z.-J., Martin J.A., Stewart A.F., Gobira P.H., Iida M., An C., Cobb M.M. (2020). Neuroadaptations in the dorsal hippocampus underlie cocaine seeking during prolonged abstinence. Proc. Natl. Acad. Sci. USA.

[24] Badisa R.B., Kumar S.S., Mazzio E., Haughbrook R.D., Allen J.R., Davidson M.W., Fitch-Pye C.A., Goodman C.B. (2015). N-Acetyl Cysteine Mitigates the Acute Effects of Cocaine-Induced Toxicity in Astroglia-Like Cells. PLoS ONE.

[25] Smith M.L., Shimomura E., Paul B.D., Cone E.J., Darwin W.D., Huestis M.A. (2010). Urinary excretion of ecgonine and five other cocaine metabolites following controlled oral, intravenous, intranasal, and smoked administration of cocaine. J. Anal. Toxicol..

[26] Jones A.W., Holmgren A. (2013). Concentrations of Cocaine and Benzoylecgonine in Femoral Blood from Cocaine-Related Deaths Compared with Venous Blood from Impaired Drivers. J. Anal. Toxicol..

[27] Pandhare J., Addai A.B., Mantri C.K., Hager C., Smith R.M., Barnett L., Villalta F., Kalams S.A., Dash C. (2014). Cocaine en-hances HIV-1–induced CD4 T-Cell apoptosis: Implications in disease progression in cocaine-abusing HIV-1 patients. Am. J. Pathol..

[28] Mittleman R.E., Wetli C.V. (1984). Death caused by recreational cocaine use: An update. JAMA.

[29] Del Olmo-Turrubiarte A., Calzada-Torres A., Diaz-Rosas G., Lara I.P., Sánchez-Urbina R., Balderrábano-Saucedo N., González-Márquez H., Garcia-Alonso P., Contreras-Ramos A. (2015). Mouse models for the study of postnatal cardiac hypertrophy. IJC Heart Vasc..

[30] Huang C.L.-H. (2017). From channels to systems: Ca2+ -sensitive K+ currents, alternans and cardiac arrhythmia. J. Physiol..

[31] Welder A., Smith M., Ramos K., Acosta D. (1988). Cocaine-induced cardiotoxicity in vitro. Toxicol. In Vitro.

[32] Chen C.W.R., Makkiya M., Aronow W., Spevack D.M. (2020). Heightened risk of cardiac events following percutaneous coronary intervention for cocaine-associated myocardial infarction. Arch. Med. Sci..

[33] Bachi K., Mani V., Jeyachandran D., Fayad Z.A., Goldstein R.Z., Alia-Klein N. (2017). Vascular disease in cocaine addiction. Atherosclerosis.

[34] Xiao Y., He J., Gilbert R.D., Zhang L. (2000). Cocaine induces apoptosis in fetal myocardial cells through a mitochondria-dependent pathway. J. Pharmacol. Exp. Ther..

[35] Sinha-Hikim I., Shen R., Nzenwa I., Gelfand R., Mahata S.K., Sinha-Hikim A.P. (2011). Minocycline suppresses oxidative stress and attenuates fetal cardiac myocyte apoptosis triggered by in utero cocaine exposure. Apoptosis.

[36] Álvaro-Bartolomé M., La Harpe R., Callado L.F., Meana J.J., García-Sevilla J. (2011). Molecular adaptations of apoptotic pathways and signaling partners in the cerebral cortex of human cocaine addicts and cocaine-treated rats. Neuroscience.

[37] Jonkman J., Cathcart J.A., Xu F., Bartolini M.E., Amon J.E., Stevens K.M., Colarusso P. (2014). An introduction to the wound healing assay using live-cell microscopy. Cell Adhes. Migr..

[38] Itou J., Oishi I., Kawakami H., Glass T.J., Richter J., Johnson A., Lund T.C., Kawakami Y. (2012). Migration of cardiomyocytes is essential for heart regeneration in zebrafish. Development.

[39] Carrillo X., Curós A., Muga R., Serra J., Sanvisens A., Bayes-Genis A. (2011). Acute coronary syndrome and cocaine use: 8-year prevalence and inhospital outcomes. Eur. Heart J..

[40] Song S.H., Park K., Kim S.W., Paick J., Cho M.C. (2015). Involvement of Rho-Kinase/LIM Kinase/Cofilin Signaling Pathway in Corporal Fibrosis after Cavernous Nerve Injury in Male Rats. J. Sex. Med..

[41] Cui K., Luan Y., Wang T., Zhuan L., Rao K., Wang S., Ye Z.-Q., Liu J.-H., Wang D.-W. (2016). Reduced corporal fibrosis to protect erectile function by inhibiting the Rho-kinase/LIM-kinase/cofilin pathway in the aged transgenic rat harboring human tissue kallikrein. Asian J. Androl..

[42] Kligys K., Claiborne J.N., DeBiase P.J., Hopkinson S.B., Wu Y., Mizuno K., Jones J.C.R. (2007). The Slingshot Family of Phosphatases Mediates Rac1 Regulation of Cofilin Phosphorylation, Laminin-332 Organization, and Motility Behavior of Keratinocytes. J. Biol. Chem..

[43] Toda S., Shen H.-W., Peters J., Cagle S., Kalivas P.W. (2006). Cocaine Increases Actin Cycling: Effects in the Reinstatement Model of Drug Seeking. J. Neurosci..

[44] Yao H., Kim K., Duan M., Hayashi T., Guo M.-L., Morgello S., Prat A., Wang J., Su T.-P., Buch S. (2011). Cocaine Hijacks 1 Receptor to Initiate Induction of Activated Leukocyte Cell Adhesion Molecule: Implication for Increased Monocyte Adhesion and Migration in the CNS. J. Neurosci..

[45] Ali M., Heyob K., Tipple T., Pryhuber G.S., Rogers L.K. (2018). Alterations in VASP phosphorylation and profilin1 and cofilin1 expression in hyperoxic lung injury and BPD. Respir. Res..

[46] Okayama T., Kikuchi S., Ochiai T., Ikoma H., Kubota T., Ichikawa D., Fujiwara H., Okamoto K., Sakakura C., Sonoyama T. (2008). Attenuated response to liver injury in moesin-deficient mice: Impaired stellate cell migration and decreased fibrosis. Biochim. Biophys. Acta -Mol. Basis Dis..

[47] Arpin M., Chirivino D., Naba A., Zwaenepoel I. (2011). Emerging role for ERM proteins in cell adhesion and migration. Cell Adhes. Migr..

[48] Van Eldik W., Adel B.D., Monshouwer-Kloots J., Salvatori D., Maas S., Van Der Made I., Creemers E., Frank D., Frey N., Boontje N. (2017). Z-disc protein CHAPb induces cardiomyopathy and contractile dysfunction in the postnatal heart. PLoS ONE.

[49] Kim W., Shin S., Kim S., Jeon S., Kim J.-H. (2009). Cocaine regulates ezrin–radixin–moesin proteins and RhoA signaling in the nucleus accumbens. Neuroscience.

[50] Sartoretto J.L., Jin B.Y., Bauer M., Gertler F.B., Liao R., Michel T. (2009). Regulation of VASP phosphorylation in cardiac myocytes: Differential regulation by cyclic nucleotides and modulation of protein expression in diabetic and hypertrophic heart. Am. J. Physiol. Circ. Physiol..

[51] Schlegel N., Waschke J. (2009). VASP is involved in cAMP-mediated Rac 1 activation in microvascular endothelial cells. Am. J. Physiol. Physiol..

[52] Brahmbhatt A.A., Klemke R.L. (2003). ERK and RhoA Differentially Regulate Pseudopodia Growth and Retraction during Chemotaxis. J. Biol. Chem..

[53] Scott M.G.H., Pierotti V., Storez H., Lindberg E., Thuret A., Muntaner O., Labbé-Jullié C., Pitcher J.A., Marullo S. (2006). Cooperative Regulation of Extracellular Signal-Regulated Kinase Activation and Cell Shape Change by Filamin A and β-Arrestins. Mol. Cell. Biol..

[54] Mendoza M.C., Er E.E., Zhang W., Ballif B.A., Elliott H.L., Danuser G., Blenis J. (2011). ERK-MAPK Drives Lamellipodia Protrusion by Activating the WAVE2 Regulatory Complex. Mol. Cell.

[55] Schwope D.M., Karschner E.L., A Gorelick D., A Huestis M. (2011). Identification of Recent Cannabis Use: Whole-Blood and Plasma Free and Glucuronidated Cannabinoid Pharmacokinetics following Controlled Smoked Cannabis Administration. Clin. Chem..

[56] Karschner E.L., Schwilke E.W., Lowe R.H., Darwin W.D., Herning R.I., Cadet J.L., Huestis M.A. (2009). Implications of Plasma 9-Tetrahydrocannabinol, 11-Hydroxy-THC, and 11-nor-9-Carboxy-THC Concentrations in Chronic Cannabis Smokers. J. Anal. Toxicol..

[57] Fabritius M., Favrat B., Chtioui H., Battistella G., Annoni J.-M., Appenzeller M., Dao K., Fornari E., Lauer E., Mall J.-F. (2013). THCCOOH concentrations in whole blood: Are they useful in discriminating occasional from heavy smokers?. Drug Test. Anal..

[58] Okada H., Lai N.C., Kawaraguchi Y., Liao P., Copps J., Sugano Y., Okada-Maeda S., Banerjee I., Schilling J.M., Gingras A.R. (2013). Integrins protect cardiomyocytes from ischemia/reperfusion injury. J. Clin. Investig..

[59] Shewchuk L.J., Bryan S., Ulanova M., Khaper N. (2010). Integrin β3 prevents apoptosis of HL-1 cardiomyocytes under conditions of oxidative stress. Can. J. Physiol. Pharmacol..

[60] Cerretani D., Fineschi V., Bello S., Riezzo I., Turillazzi E., Neri M. (2012). Role of oxidative stress in cocaine-induced cardiotoxicity and cocaine-related death. Curr. Med. Chem..

[61] Chi Z., Melendez A.J. (2007). Role of Cell Adhesion Molecules and Immune-Cell Migration in the Initiation, Onset and Development of Atherosclerosis. Cell Adhes. Migr..

[62] Patrizi R., Pasceri V., Sciahbasi A., Summaria F., Rosano G.M., Lioy E. (2006). Evidence of Cocaine-Related Coronary Atherosclerosis in Young Patients With Myocardial Infarction. J. Am. Coll. Cardiol..

[63] Ambrose J.A., Singh M. (2015). Pathophysiology of coronary artery disease leading to acute coronary syndromes. F1000Prime Rep..

[64] Kumar V., Gopalakrishnan L., Singh M., Singh S., Kovacs D.F., Benatar D., Gibson C.M., Khosla S. (2018). Effect of cocaine on coronary microvasculature. J. Am. Coll. Cardiol..

[65] Kim S.T., Park T. (2019). Acute and Chronic Effects of Cocaine on Cardiovascular Health. Int. J. Mol. Sci..

[66] Witek P., Korga A., Burdan F., Ostrowska-Lesko M., Nosowska B., Iwan M., Dudka J. (2016). The effect of a number of H9C2 rat cardiomyocytes passage on repeatability of cytotoxicity study results. Cytotechnology.

[67] Borini P., Guimarães R.C., Borini S.B. (2004). Possible hepatotoxicity of chronic marijuana usage. Sao Paulo Med. J..

