# Supplementary material for: Metabolites of Cannabis Induce Cardiac Toxicity and Morphological Alterations in Cardiac Myocytes
Source: Int J Mol Sci. 2022 Jan 26;23(3):1401. doi: 10.3390/ijms23031401 (PMC8835806; doi:10.3390/ijms23031401)
Supplement: Supplementary file 1 [file ijms-23-01401-s001.zip › ijms-1562220-supplementary.pdf]

International Journal of
Molecular Sciences

MDPI

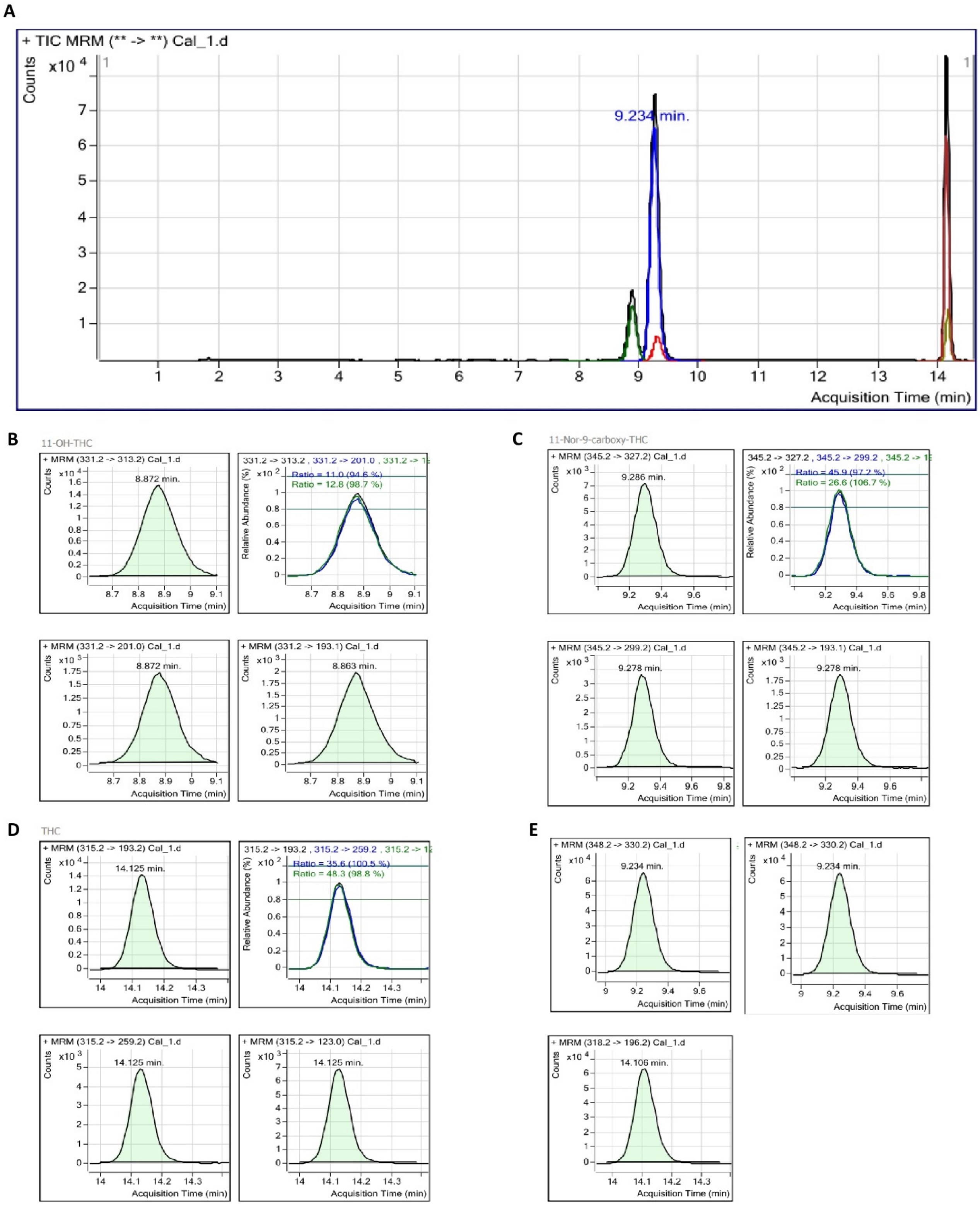

Figure S1. Total Ion Chromatogram of Multiple Reaction Monitoring chromatograms for cannabinoid analysis. Limit of quantification sample (Cal 1) used as a representative of peak resolution and separation. **(A)** Total ion chromatogram and overview of chromatographic run with retention times for THC-OH, THC-COOH and THC. Individual MRM chromatograms displaying three transitions and ion ratio acceptance for (**B**) THC-OH, (**C**) THC-COOH, (**D**) THC and (**E**) single ion transitions for the deuterated internal standards.
